# A prospective analysis of falls in Parkinson's disease: Does physical capacity moderate the relationship between walking amount and falls rates?

**DOI:** 10.1177/1877718X261418987

**Published:** 2026-02-13

**Authors:** Ríona Mc Ardle, Lisa Alcock, Heather Hunter, Brook Galna, Alan Godfrey, Rachael A Lawson, Silvia Del Din, Alison Yarnall, Jochen Klenk, Lynn Rochester, on behalf of the ICICLE-Gait team

**Affiliations:** 1 Translational and clinical research institute, Faculty of Medical Sciences, Newcastle University, Newcastle Upon Tyne, NE4 5PL, UK; 2 NIHR Newcastle Biomedical Research Centre, Newcastle University and The Newcastle upon Tyne Hospitals NHS Foundation Trust, Newcastle upon Tyne, UK; 3 Clinical Ageing Research Unit, The Newcastle Upon Tyne Hospitals NHS Foundation Trust, Newcastle Upon Tyne, UK; 4 School of Allied Health (Exercise Science), Murdoch University, Perth, Western Australia, Australia; 5 Centre for Healthy Ageing, Health Futures Institute, Murdoch University, Perth, Western Australia, Australia; 6 School of Computer Science, Northumbria University, Newcastle upon Tyne, NE1 8ST, UK; 7 Institute of Epidemiology and Medical Biometry, Ulm University, Ulm, Germany; 8 Study Center Stuttgart, IB University of Health and Social Sciences, Stuttgart, Germany

**Keywords:** Parkinson's disease, falls, walking, longitudinal study

## Abstract

Falls are a significant concern for people with Parkinson's disease (PwP), often leading to restriction of walking activities to avoid situations where falls may occur. However, limited research has explored the relationship between walking amount (i.e., daily steps) and falls, particularly how this relationship may be influenced by physical capacity (i.e., gait speed). This study aimed to address that gap. Results indicate that higher daily step counts were associated with higher fall rates in PwP with moderate physical capacity, and lower fall rates in those with high capacity. The relationship between walking and fall rates was moderated by physical capacity.

## Plain language summary

Rethinking the relationship between walking and falls in Parkinson's: One size doesn’t fit all

People with Parkinson's often fall. Those falls can cause injuries, make people fearful of falling again, lowering their quality of life, increasing stress for carers, and meaning they need more healthcare support. Many people with Parkinson's and their carers think that walking more could increase the chance of falling. As a result, they sometimes choose to walk less. Very few studies have looked at the link between walking activity and falls in Parkinson's. This study aimed to understand if walking more might lead to more falls in Parkinson's, while considering how a person's physical capacity (i.e., a person's ability to carry out physical activities) might play a role in that link.

To explore this, we recruited 84 people with early-stage Parkinson's to take part in a study which involved assessments every 18 months. At the first visit, they did several clinical exams, including a walking assessment in which we asked them to walk at their own usual pace, from which we measured their physical capacity. They also wore a small sensor on their thigh for up to seven days, which counted how many steps they took each day. Participants filled in a diary every month for up to three years to tell us how many times they had fallen, if at all.

We found that walking on its own was not linked to having more falls. Instead, the link depended on a person's physical capacity. For people in the early stages of Parkinson's who had higher physical capacity, taking more steps was associated with less falls. However, for people with lower physical capacity, taking more steps seemed to slightly increase the number of falls. Future efforts to prevent falls in Parkinson's should consider assessing a person's physical capacity first and tailoring recommendations around participating in walking activity.

## Introduction

Falls are a significant concern for people with Parkinson's (PwP), leading to injuries, diminished quality of life, and greater carer stress and healthcare needs.^1,2^ PwP are twice as likely to fall as similarly-aged older adults, and usually fall recurrently.^1,2^ Common risk factors for falls in PwP include older age, gait disturbances, prior history of falling, greater disease severity and cognitive impairment.^1–3^ Previous diary-based studies report that 45–67% of falls occur during walking in PwP, commonly due to tripping in single-time fallers and muscle weakness in recurrent fallers.^4–6^

To reduce exposure to situations in which a fall may occur, PwP may restrict their walking amount (quantity of walking in a given time, e.g., steps per day).^5,7–9^ That leads to physical deconditioning which is conversely associated with increased risk of recurrent falls. However, the extent to which a greater amount of walking contributes to higher fall rates in PwP remains unclear. Previous studies have produced inconsistent findings whereby higher falls incidence have been associated with a higher^10^ and lower walking amount^11^ or no association.^12^ Those few studies have relatively short follow-up periods (e.g., 12 months) and vary across disease stages. As walking plays a fundamental role in PwPs’ physical, mental, emotional and social wellbeing and independence, further efforts to understand the relationship between walking amount and fall rates could inform person-centred fall prevention guidance.^13^

One consideration is the potential moderating role of physical capacity in this relationship. Physical capacity is a global term for the ability to perform physical tasks using performance-based measures e.g., gait speed, muscular endurance assessments.^14^ In particular, gait speed is considered “the sixth vital sign”, a marker of functional decline,^15^ and a predictor of future falls in Parkinson's disease.^16^ Recent findings in older long-term care residents, a population who also shows significant falls risk, suggests that physical capacity level (measured by the Short Physical Performance Battery)^17^ modifies the relationship between walking amount and fall rates.^18^ Residents with low physical capacity showed higher baseline fall rates and a greater proportion experienced injurious falls, but daily step count was not associated with fall rates in that group.^18^ However, those with moderate physical capacity had lower baseline fall rates and fewer individuals with fall-related injuries, but fall rates moderately increased with a higher step count, without a corresponding rise in injurious fallers. That highlights the need to consider how physical capacity and walking amount interact and contribute to falls, as stratification may support design of effective falls prevention interventions and clinical recommendations. However, long-term care residents are a frail co-morbid population with environmental and policy-level restrictions impacting their walking amount,^19–22^ not directly comparable to community-dwellers with Parkinson's disease. Yet, that interaction has not been explored in PwP. To address the gap, this exploratory proof-of-concept study aimed to examine whether physical capacity moderates the relationship between walking amount (i.e., baseline daily step count) and fall rates over three years in early-stage, community-dwelling PwP.

## Methods

### Participants

Eligible participants were enrolled in the Incidence of Cognitive Impairment in Cohorts with Longitudinal Evaluation–Parkinson's disease (ICICLE-PD) and ICICLE-Gait studies. Full study details, including recruitment and eligibility have been previously described.^12,23,24^ The study was approved by a local NHS Research Ethics Committee and all participants gave written informed consent. Participants were included in this analysis if they had completed a baseline real-world activity monitoring assessment for ≥3 consecutive days, a lab-based walking assessment, and provided falls diaries during the 3-year assessment period.

### Demographic and clinical variables

All participants underwent a comprehensive clinical assessment at baseline.^23^ Motor disease severity and disease stage were assessed using the Movement Disorders Society Unified Parkinson's Disease Rating Scale III (MDS-UPDRS III) and Hoehn & Yahr.^25,26^ Global cognition was assessed using the Mini Mental State Examination.^27^ Gait speed was determined during a two-minute walk at participants’ preferred pace around a 25-metre circuit using a 7-metre instrumented walkway (GAITRite, CIR Systems Inc., 240 Hz).^28,29^ Gait speed was calculated across all recorded steps per person using the following equation:

$$\frac{\left(\frac{Mean\;length\;of\;all\;left\;steps}{Mean\;duration\;of\;all\;left\;steps}\right)+\left(\frac{Mean\;length\;of\;all\;right\;steps}{Mean\;duration\;of\;all\;right\;steps}\right)}{2}$$

Self-selected gait speed, a recognised measure of physical capacity and strongly correlated with the Short Physical Performance Battery, was used to characterise participants as “high” (≥1 metre/second) or “moderate” physical capacity (<1 metre/second).^30^ Those cut-points align with recognised thresholds to identify older adults at high risk of adverse health events.^31^ Although <0.8 m/s is commonly considered “low” physical capacity, participants were not classified in a separate group due to the limited number (n = 4) but retained in the moderate group.^31^

### Real-world activity monitoring

Activity data were collected at baseline using a validated activPAL™ activity monitor (20 g, 10 Hz, 15-s epochs), worn on the upper thigh for up to seven days.^32^ The primary outcome was daily step count.

### Fall rates

Retrospective faller status was identified by self-report of a fall within the previous year of baseline. Prospective falls were recorded using standardised falls diaries sent out monthly and any gaps in data clarified by telephone^33^; our analysis used diaries collected within 36-months from baseline. Fall rates were calculated as number of falls per year. A maximum value of 10 was assigned to total number of falls to limit effects of extreme variance in statistical models.^34^

### Data analysis

Between-group comparisons for demographics were assessed using Mann-Whitney U tests and Pearson's chi-square test for continuous and categorical variables respectively. A general linear model with a quasi-poisson family was used to examine the interaction between physical capacity level and steps taken per day in determining fall rates during the study, offset for observation time; this model included motor disease severity (MDS-UPDRS III), body mass index (BMI) and retrospective faller status as covariates. The ratio of participants (n = 84) to parameters (n = 6) was considered adequate based on the commonly used rule of thumb of approximately 10 participants per parameter in linear regression.^35^ All recorded prospective falls during the time period were included in analysis (capped at 10 falls per person), with fall count as the dependent variable within the model, allowing the modelling of the full spectrum of fall frequence including those who experienced recurrent falls. Coefficients were presented as log estimates (β), which were exponentiated to obtain rate ratios and 95% confidence intervals (CI) to support interpretability. A rate ratio of >1 indicates an increase while <1 suggest a decrease of daily step count on fall rates.

## Results

121 participants were recruited to ICICLE-Gait, with 84 retained for this analysis. Exclusion from this analysis was due to no follow-up visit within the 36-month observation period (n = 14) and not completing baseline real-world activity monitoring (n = 24), both essential components for the analysis. Mean duration in the study was 32 ± 8 months. At baseline, 19% were characterised as retrospective fallers, while 56% reported falling during the observation period. Baseline demographics and fall rates are reported in Table 1.

**Table 1.** Baseline demographic and clinical characteristics of the sample.

	All N = 84	High physical capacity N = 63 (75%)	Moderate physical capacity N = 21 (25%)	p-value
Age (years), mean (SD)	67 (10)	67 (10)	68 (10)	0.549
Sex, n (%)				0.225
Female	27 (32%)	18 (29%)	9 (43%)	
Male	57 (68%)	45 (71%)	12 (57%)	
BMI (kg/m^2^), mean (SD)	27.5 (4.5)	26.8 (4.2)	29.6 (4.6)	**0**.**028**
Education (years), mean (SD)	13.2 (3.8)	13.5 (3.9)	12.4 (3.4)	0.261
Time since PD diagnosis (months), mean (SD)	6.4 (5.0)	6.5 (5.2)	6.3 (4.4)	0.946
MDS-UPDRS-III score (n/132), mean (SD)	25 (11)	23 (11)	30 (9)	**0**.**005**
Hoehn & Yahr stage (I-IV), n (%)				**<0**.**001**
I	20 (24%)	18 (29%)	2 (9.5%)	
II	49 (58%)	40 (63%)	9 (43%)	
III	15 (18%)	5 (7.9%)	10 (48%)	
MMSE score (n/30), mean (SD)	28.65 (1.24)	28.70 (1.16)	28.52 (1.47)	0.851
Gait speed (m/s), mean (SD)	1.14 (0.19)	1.22 (0.13)	0.90 (0.11)	**<0**.**001**
Steps per day, mean (SD)	5710 (2465)	5998 (2548)	4849 (2013)	**0**.**046**
Activity monitoring time (days), mean (SD)	6.77 (0.79)	6.71 (0.83)	6.94 (0.62)	0.926
Observation time (months), mean (SD)	32 (8)	32 (8)	31 (8)	0.861
Retrospective faller, n (%)				**0**.**021**
Yes	16 (19%)	8 (13%)	8 (38%)	
No	68 (81%)	55 (87%)	13 (62%)	
Faller during observation, n (%)				**0**.**002**
Non-Faller	37 (44%)	34 (54%)	3 (14%)	
Study Faller	47 (56%)	29 (46%)	18 (86%)	
Falls per person-year	0.87 (1.52)	0.62 (1.35)	1.60 (1.77)	**<0**.**001**

Abbreviations: BMI = body mass index; PD = Parkinson's disease; MDS-UPDRS = Movement Disorders Society Unified Parkinson's disease rating scale; MMSE = Mini mental state examination.

Based on the described Gait speed thresholds, participants were categorised as having high (n = 63 (75%)) or moderate (n = 21 (25%)) physical capacity. Group differences are reported in Table 1.

### Effect of daily step count on fall rates differs by physical capacity level

**Figure 1.**
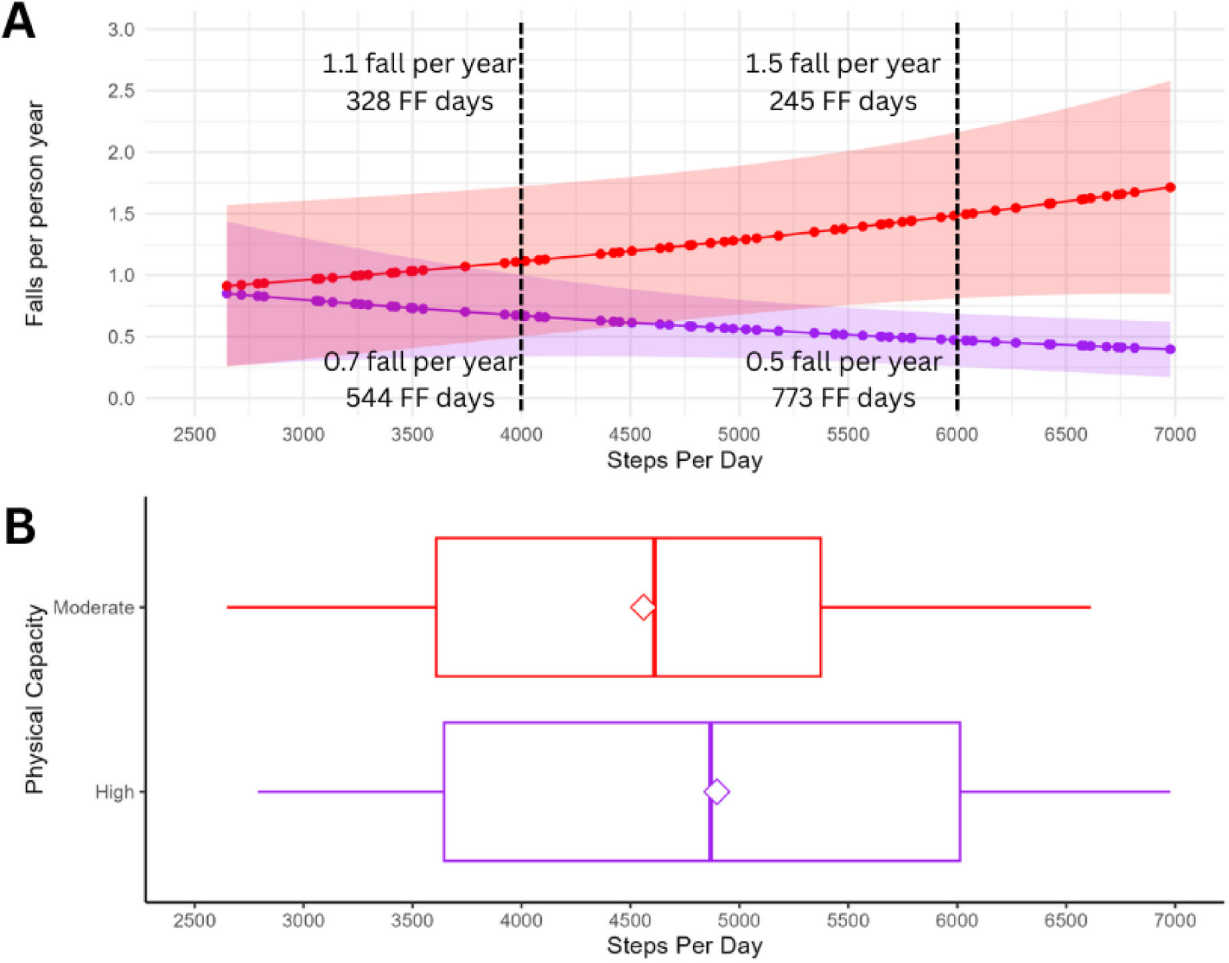
The interaction effect between physical capacity levels and daily step count for people with Parkinson's, marked with the estimated number of falls free days and relative risk of falling across activity levels within physical capacity groups. A. Interaction plot between daily step count and physical capacity level on determining fall rates per year. Falls per year refer to model-predicted falls based on an interaction between physical capacity and step count, estimated without covariates to illustrate group-level trends for discrete activity levels (4000 and 6000 steps per day). Falls-free walking was calculated by dividing annual step amount by predicted falls. Falls-free days were derived by dividing falls-free steps by daily step count. B. Boxplot to demonstrate distribution of daily step count data across physical capacity levels. FF = falls free; * compared to those taking 4000 steps per day. ◇ = mean score.

The main effect of daily steps (*p* *=* *0.15*) and physical capacity (*p* *=* *0.25*) were not significantly associated with fall rates. There was a significant interaction, where baseline physical capacity significantly moderated the association between daily step count and fall rate (*p* *=* *0.026*; rate ratio = 1.41, 95% CI 1.05–1.92; Figure 1). PwP with moderate physical capacity fell at a 20% higher rate per additional 1000 daily steps (rate ratio: 1.20, 95% CI: 0.97 to 1.48), while those with high capacity fell at a 15% lower rate per additional 1000 daily steps (rate ratio: 0.85, 95% CI: 0.66 to 1.03).

## Discussion

This study aimed to examine how physical capacity influences the relationship between walking amount and fall rates in PwP. Building on earlier work in long-term care settings, our findings suggest that physical capacity may play a moderating role in how walking amount relates to falls in high fall-risk populations.^18^ While results should be interpreted with caution in this exploratory study, these initial insights may be useful in better understanding the relationship between walking and falls in populations with high-fall risk, such as PwP.

We therefore hypothesise that the association between walking amount and fall rates may be non-linear; in individuals with low physical capacity (as in long-term care), increased amount is not clearly linked to higher fall rates, but in those with higher capacity (as seen here), it may be protective against falls.^18^ Additionally, those with moderate physical capacity may form a key target group for falls interventions, as they have an elevated, but perhaps modifiable, fall risk potentially related to walking amount (as seen in both studies) or its influences.^18,36^ These results somewhat reflect previous results from Canning,^37^ which found that an intervention targeting balance, leg strength and freezing of gait had differential effects on PwP moderated by motor disease severity in a RCT. In milder disease severity, the exercise intervention reduced falls, but for those with greater motor disease severity in the intervention group, there was a trended increase in fall rates and proportion of fallers.^37^ Although UPDRS-III was included as a co-variate in our analysis, the moderate capacity group showed higher UPDRS-III and Hoehn and Yahr scores at a group level, which suggests the observed pattern may be comparable to these prior results.^37^ Further work is needed to replicate and extend these findings. This should include data on falls-related injuries, to allow us to understand the balance between safety with walking amount and the independence that comes with it.^38^

These “one size doesn’t fit all” findings highlight the complex nature of walking behaviours and their interaction with falls. Walking behaviours reflect a dynamic interplay between a person's physical, cognitive and affective wellbeing with their environment and the task itself.^13,39^ Therefore, our finding that greater walking amounts in PwP with moderate physical capacity are associated with higher fall rate may not be attributable to walking alone, but instead reflect the broader complexity of features that contribute to mobility.^39,40^ Physical capacity, in itself, plays an important role in mobility, and an established predictor of falls in PwP.^1,6,41^ Strength-based interventions have demonstrated reductions of fall rates up to 85%, suggesting physical capacity is also a modifiable target.^42^ Additionally, in individuals with moderate capacity, increased walking may simply raise exposure to situations in which falls are more likely to occur due to lower physical capacity, such as transfers.^12,41^ Non-motor symptoms, including cognitive impairment, executive dysfunction, anxiety, apathy and depression, can also decrease walking behaviour leading to physical deconditioning^40^ and increase fall risk independently of walking exposure.^43–45^ Non-motor symptoms also occur more commonly with greater motor disease severity in Parkinson's, reflective of our moderate physical capacity group.^46^ Further research should consider the differential effect of fall prevention interventions in discrete physical capacity groups and mediating role of common non-motor symptoms in the relationship between walking amount and falls in PwP.

Strengths of this study include use of objective activity monitoring and “gold-standard” prospective falls diaries,^47^ inclusion of relevant clinical covariates and an extended follow-up period. This study was limited by a small sample which may reduce statistical power, and while disease severity was controlled for, other potential confounders (e.g., medication, co-morbidities, non-motor symptoms) were not.^3^ Additionally, those who fell very frequently were asked to stop completing diaries, although our cap of ten falls applied during analysis may mitigate this. Falls diaries did not include a question about fall-related injuries; while some participants recorded injuries in their diaries, these reports are likely incomplete and reliable injury rates could not be extracted for analysis. Further work is required to validate our new hypotheses in a large, well-characterised cohort of PwP using both physical capacity measures employed here and by Mc Ardle^18^ (gait speed, Short Physical Performance Battery) to determine whether they produce comparable results and to assess the relative utility of each measure, and should consider including fall-related injury data in analysis. Finally due to the historical nature of this study, PwP were not involved in the design, conduct or analysis of this work; we recommend future research in this area incorporates the patient voice.

To conclude, this study provides novel evidence that the relationship between walking amount and falls differs according to physical capacity level in PwP, suggesting further investigation in a larger, well-characterised cohort is warranted.
